# The journal El Practicante Oscense (1935-1940) as a source for the study of the history of nursing during the Spanish Civil War

**DOI:** 10.1590/S0104-59702025000100027

**Published:** 2025-06-16

**Authors:** Raúl Expósito-González, Juan Carlos Muñoz Camargo, Alberto Bermejo-Cantarero, María Laura Parra-Fernández, María Dolores Onieva-Zafra, Julián Rodríguez-Almagro

**Affiliations:** i Adjunct professor, Faculty of Nursing/University of Castilla La-Mancha. Ciudad Real – Spain; raul.egonzalez@uclm.es; ii Adjunct Professor, Faculty of Nursing/University of Castilla La-Mancha. Ciudad Real – Spain; juancarlos.munoz@uclm.es; iii Assistant Professor, Faculty of Nursing/University of Castilla La-Mancha. Ciudad Real – Spain; alberto.bermejo@uclm.es; iv Associate Professor, Faculty of Nursing/University of Castilla La-Mancha. Ciudad Real – Spain; marialaura.parra@uclm.es; v Associate Professor, Faculty of Nursing/University of Castilla La-Mancha. Ciudad Real – Spain;mariadolores.onieva@uclm.es; vi Associate Professor, Faculty of Nursing/University of Castilla La-Mancha. Ciudad Real – Spain; julianj.rodriguez@uclm.es

**Keywords:** Practicantes, Male medical and surgical assistants, Professional associations, Nursing publications, Spanish Civil War, Assistente médico, Homens assistentes médicos e cirúrgicos, Associações profissionais, Publicações de enfermagem, Guerra Civil Espanhola

## Abstract

Health professional publications have proved to be a valuable source of historical research. This study analyses the journalistic life of *El Practicante Oscense*, the bulletin of the Official College of Practicantes of Huesca and its province (Colegio Oficial de Practicantes de Huesca y su provincia), published between 1935 and 1940, copies of which are kept at the Huesca Official College of Nursing (Colegio Oficial de Enfermería de Huesca). Issues such as the professional identity of *practicantes* (male medical and surgical assistants), their professional interactions with nurses, labour demands, and political, social and professional synergies are analysed, especially the collegial organization of the profession during the Spanish Civil War (1936-1939).

## Resumo

Publicações de profissionais de saúde têm se mostrado fontes valiosas para a pesquisa histórica. Este estudo analisa a vida do periódico El Practicante Oscense, boletim do Colegio Oficial de Practicantes de Huesca e província, publicado entre 1935 e 1940, cujas cópias estão guardadas no Colegio Oficial de Enfermería de Huesca. Questões como a identidade professional dos practicantes (homens assistentes médicos e cirúrgicos), suas interações profissionais com profissionais da enfermagem, suas demandas laborais, e suas sinergias políticas, sociais e profissionais são analisadas, particularmente a organização colegiada no curso da Guerra Civil Espanhola (1936-1939).

The publications produced by organizations of health professionals are a valuable source of historical research (Alarcão, Mota, 2021; Sophia, 2013). The journals published by Spanish nursing professionals, however they were designated throughout history – *practicantes* (male medical and surgical assistants), midwives, and nurses – served as a mouthpiece for their collective claims. The wealth of articles, news stories, denunciations, iconography, etc. published in their pages constitutes a very important source for constructing a historical account of the nursing profession.

Until 1953, auxiliary medical professionals working in Spain were divided into three categories: *practicantes*, midwives, and nurses. This was put to an end when new legislation grouped all three under the same title of technical health assistant (*ayudante técnico sanitario*) (Spain, 29 Dec. 1953). The new term, completely unknown outside Spain, contrasted with the globally accepted term “nurse” (Martín-Espinosa, González-García, Mirón-González, 2018). Decades later, in 1977, the title of technical health assistant was abolished to make way for the new title of qualified in nursing (*diplomado en enfermería*) (Spain, 22 Aug. 1977), which was much more in line with the international denomination.

Well into the nineteenth century, responsibility for caring for the sick in Europe’s catholic countries, including Spain, had largely fallen to male and religious orders and congregations (Hernández-Martín et al., 1997). The titles of *practicante* and midwife had been created in 1857. *Practicantes* were authorized to practise the purely mechanical, subordinate aspects of surgery, which included minor procedures, vaccination, injections, teeth extractions, and podiatry. Midwifes, for their part, were authorized to assist with normal childbirths (Spain, 1861). In addition to their competencies, these professionals were also differentiated by sex: *practicantes* were men, while midwives were women. Almost sixty years later, in 1915, the state nursing degree was regulated (Spain, 1915).

One appropriate framework for addressing the object of this research is the sociology of professions, as described by Blázquez-Ornat (2016). This author argues that although there are different conceptualizations of professions, the classic Weberian definition still prevails among much of the scientific community. This definition sees a profession as an occupation that is socially idealized and organized as a collective or closed associative community that occupies a position of privilege in workforce classifications, primarily for the type of knowledge and skills its practitioners must acquire.

For Siles-González (1994), an individual’s profession is the primary means by which they engage with the economic and social system. In Spain, colleges of *practicantes*, midwives, and nurses, as instruments of control, were decisive in the social projection of their respective professions through the adoption, propagation, and implementation of functions among their members. By the early 1900s, voluntary associations and colleges of *practicantes* had proliferated in every corner of the country, along with their official bulletins. Colleges of midwives gained a similar presence from the third decade onwards. Official registration was made compulsory for *practicantes* in late 1929 and for midwives in May 1930. The National Federation of Colleges of Practicantes and the National Federation of Colleges of Midwives also formed general councils of colleges for their respective professionals (Spain, 29 Dec. 1929, 9 May 1930). Nursing was the last of these professions to have mandatory registration, which was introduced in 1944. The journals published first by the voluntary colleges and later by the official colleges became the mouthpieces for the demands of these professional groups. The articles and news items reflected their collective aspirations, concerns, and setbacks (Lasarte-Calderay, 1993), making them invaluable primary sources for researchers.

The first historians of Spanish nursing to take an interest in the early publications of these “auxiliary health professions” were working in the 1990s. Since then, research on the topic has blossomed. A catalogue has even been published that compiles a large part of the bulletins and journals produced by *practicantes*, midwives, nurses, and technical health assistants from the mid-1800s to the beginning of the reign of Juan Carlos I (1975).

As a prelude to this study, it is worth making a brief historical review of the *practicantes*’ publications, to place the magazine of interest here in its context. The first professional bulletins began to appear in the second half of the nineteenth century, spearheaded by the Madrid-based *La Voz de los Ministrantes*, whose first issue was published on Friday, January 15, 1864. The entity responsible for this publication was the Madrid Society of Bleeders, which had been in operation since 1860. The next publications to be created were, in chronological order: *El Genuino* (1865), the official bulletin of the Seville College of Bleeders and Practicantes; *El Cirujano Menor*, Madrid (1872); *La Lanceta*, official journal of the Union of Surgeons, Bleeeders, and Practicantes of Barcelona (1883); *La Voz de los Practicantes*, Morella (1884); *El Practicante*, Zaragoza (1884), affiliated to the General Association of Practicantes of Spain; *El Defensor del Practicante*, Madrid (1885), supporter of the League of Practicantes of Spain; *El Eco del Practicante*, La Coruña (1888); *El Fígaro Moderno*, Madrid (1897); and *El Practicante Español*, Arévalo de la Sierra (1899) (Expósito-González, 2009).

A large part of the publications produced by voluntary colleges and associations in the early twentieth century has been catalogued by Álvarez-Nebreda (2010). These include: *El Practicante Valenciano*, *El Practicante Moderno*, *El Practicante del Norte*, *La Cirugía Menor*, *Boletín de los Colegios de Practicantes de Medicina y Cirugía*, and *El Practicante Gaditano*.

In the province of Huesca, which, together with Zaragoza and Teruel, makes up the autonomous community of Aragón, a Provincial College of Practicantes of Medicine and Surgery was founded on September 18, 1917 (La Asamblea…, 1917). However, it was several years before it published its own bulletin. Álvarez-Nebreda (2010) refers to the existence of another journal prior to the one in question, entitled *Boletín Oficial del Colegio de Practicantes de Huesca*, which was published in approximately 1930, but no copies of which have been found. In January 1932, a regional journal, *El Auxiliar Médico Aragonés*, began to be published in the neighbouring province of Zaragoza. It carried news stories and announcements and covered other matters relating to the Huesca college, which later even gained a dedicated space in the journal. Blázquez-Ornat (2016) focused her research on the analysis of four journals published in Aragón*: El Practicante*, *El Practicante Aragonés*, *El Auxiliar Médico Aragonés*, and *El Auxiliar Médico Español*.

The purpose of this research was to analyse the trajectory of *El Practicante Oscense*, the official bulletin of Official College of Practicantes of Huesca and its province (Colegio Oficial de Practicantes de Huesca y su provincia), first published in October 1935. This was the heyday of this type of publication: in 1980, Piñero and Ferrandis (cited in Herrera-Rodríguez, 1992-1993, p.252) stated that a total of 16 health auxiliary journals were founded between 1919 and 1938. The study of the Huesca journal sheds light on the problems and concerns of the *practicantes* from the province and how their collegiate life developed. Although it began during the Second Republic (1931-1939), it was impacted directly by the Spanish Civil War (1936-1939), during which time its publication was suspended, before it reappeared with a stated adherence to the new regime.

## Sources used

The primary documental source used in this study was *El Practicante Oscense*, one of the journals published by Spanish colleges of *practicantes*. We felt it was a valid source for investigating the history of Spanish nursing during the Spanish Civil War, because although its publication was suspended after the outbreak of hostilities, it was resumed before the war reached an end. Specifically, it was first launched in October 1935 and had monthly issues until July 1936; then, after a two-year hiatus, it was relaunched in July 1938 and continued until March 1940. The Huesca nursing college, Colegio Oficial de Enfermería de Huesca, has a bound copy of the complete series, except for issue number 15.^1^ Huesca public library (Biblioteca Pública del Estado en Huesca) also has a copy of issue number 8, from May 1936.

To locate other publications of a similar nature, we consulted the historical collection at the library of the Madrid nursing college, Colegio Oficial de Enfermería de Madrid, and the digital newspaper library Biblioteca Virtual de Prensa Histórica, which is managed by the Spanish Ministry of Culture. We also consulted the historical collection of the *Gaceta de Madrid* and the *Boletín Oficial del Estado* for legislative sources. In addition, we reviewed the literature on the history of nursing, nursing publications, and the collegial organization of nursing in Spain available in the Castilla-La Mancha and Cadiz university libraries, Wiley Online Library, and the CUIDEN, Dialnet, and SciELO databases.

### Practicantes in Huesca through the lens of *El Practicante Oscense*

Some analyses of the profession of *practicante*, based on the sociology of professions, have revealed fundamental aspects of its conceptualization. These include *practicantes*’ professional identity, the technical discourse of authorities, their professional interactions with nurses (including gender inequality), professional issues, explicit claims, and political social and professional synergies (Blázquez-Ornat, 2016). Our study is divided into two parts. The first presents an analysis of the journal’s structure, as well as its trajectory and main features. The second part focuses on its contents, particularly the most important aspects for the professional development of the *practicantes* in Huesca during the period under study, with special attention to the information on the collegial organization of the profession during the civil war.

### Structure of the journal

#### Aim

The editorial of the first issue of *El Practicante Oscense*, promoted by the Official College of Practicantes of Huesca, made its purpose clear: to defend the interests of *practicantes*, especially those working in rural areas, as the college’s membership was largely rural. The journal maintained this editorial line throughout its existence. Created in the image and likeness of the journals of other colleges being published at the time, *El Practicante Oscense* was the ideal way for the Huesca college to promote its work and develop stronger union with the other colleges (Redacción, 1935).

#### Evolution

**Figure 1:**
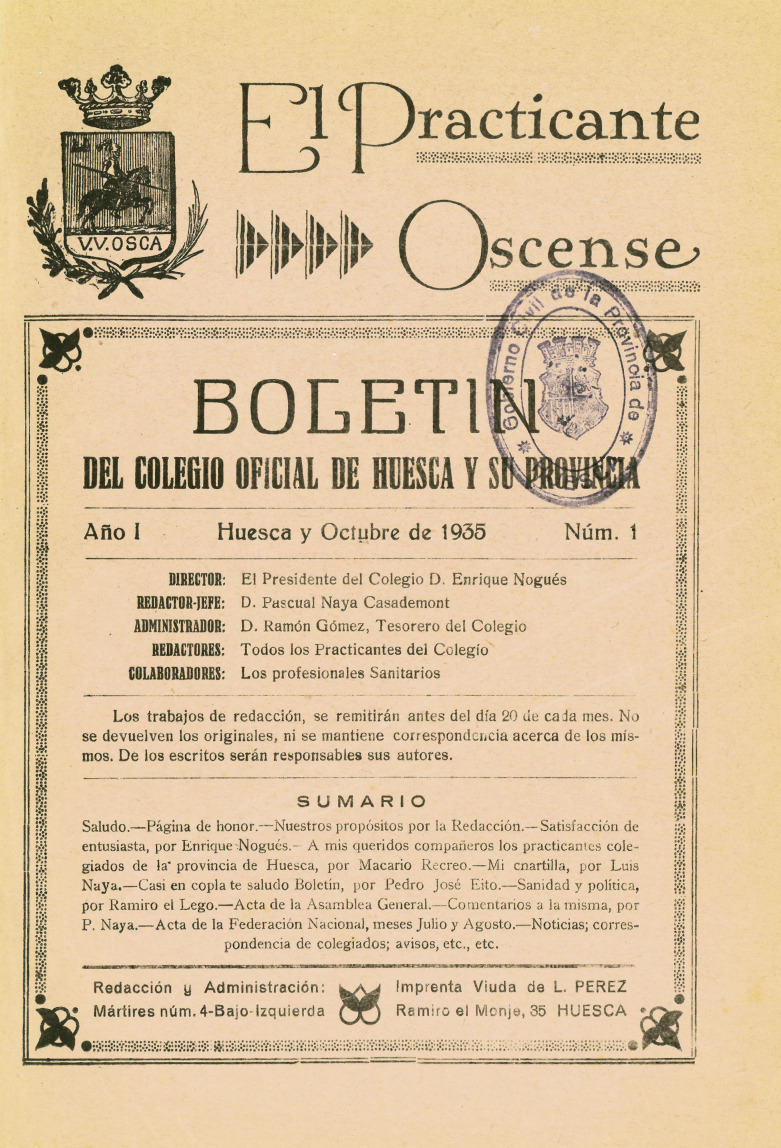
: Cover of the first issue of the journal *El Practicante Oscense*, 1935 (Source: Colegio Oficial de Enfermería de Huesca)

*El Practicante Oscense* (Figure 1) was founded in October 1935, during the Second Republic. Its first editor was Enrique Nogués, who was president of the Official College of Practicantes of Huesca. The editor-in-chief was Pascual Naya Casademont, and the administrator was Ramón Gómez Gil, who held the positions of secretary and treasurer within the corporation. The journal, whose subtitle was “Bulletin of the Official College of Huesca and its Province,” was published monthly in its first period. The editorial office and administration were located on the first floor (left) of number 4, Calle Las Mártires, and it was printed a few meters away at Imprenta de la Viuda de L. Pérez (“printing house of the widow of Pérez”), at number 35, Calle Ramiro el Monje. We were unable to ascertain how many copies were printed, or how much was charged for subscription to the journal or the purchase of a single issue. The journal was printed in quarto format with covers. Each issue generally had 16 pages, but there were some exceptions: the first issue had 27 pages; issue number 6 (March 1936) was published as an extraordinary issue with 40 pages; and issue number 10 (July 1936) – the last in the first phase of publication – had 20 pages. It was common for this type of publication not to have a fixed number of pages. All the registered *practicantes* from the region were listed as editors, and the physicians were listed as collaborators.

After the outbreak of civil war on July 18, 1936, *El Practicante Oscense* suspended its publication. Issue number 11 came out two years after issue number 10, in July 1938, marking the beginning of the second phase of the journal. The front and back covers and the format remained the same as before, but there were fewer pages. Most of the issues now had just eight pages, but, as in the first period, there were exceptions: issues number 11 and number 28 had 12 pages; issues number 20, 21, and 25 had ten pages; and issue number 26 had six pages. The journal maintained practically the same structure and contents, but its editorial line was now clearly aligned with the rebel faction, with the pages of all its issues bearing slogans in favour of the rebel troops and their leader, General Francisco Franco Bahamonde. In this second period, responsibility for health was transferred to the Ministry of the Interior, instead of the Ministry of Health, created during the Second Republic. The new director of the journal was the president of the college, Pascual Naya, while Ramón Gómez continued as its administrator. The editors were the members of the governing board of the college and the collaborators were “all colleagues.” Later, the word “colleagues” was replaced by “*practicantes*.” The journal’s subtitle was barely altered. In 1939 and 1940 the journal continued to be published monthly, with occasional exceptions, when it was published every two months. The editorial office and administration occupied the same address, but the journal was now printed by Falange Española Tradicionalista y de las Juntas de Ofensiva Nacional-Sindicalista (FET de las JONS). This was the name of the new single political party founded on April 19, 1937, in the middle of the civil war, merging the fascist party, Falange Española de las JONS, with the Carlist Party, maximum exponents of the coup d’état perpetrated by the army that started the Spanish Civil War (Tuñón de Lara, García-Nieto, 1992). FET de las JONS was the party of the Franco regime, installed after the civil war, and formed part of the machinery of the new state, known as the Movimiento Nacional (National Movement). From issue number 16 (December 1938) on, the journal was published by the printing works of *Nueva España*, a National Movement newspaper founded in Huesca in November 1936 (Arco, 1952). As in its first stage, we were unable to ascertain the size of each print run, the subscription prices, and the price of single issues. The journal ceased to be published after March 1940 due to the paper shortage in the postwar period (Arco, 1952).

## Principal features of the journal

Although *El Practicante Oscense* had a very short lifespan, its structure is not easy to analyse. Some sections are clearly defined in the list of contents, although they may not be present in all issues; other sections are not listed as such, but their labels can be deduced from their contents. For greater clarity, the information has been grouped into different sections: editorial, official matters (laws, regulations), professional matters (minutes of meetings), scientific matters, current affairs (opinion articles), financial matters (collegial budgets), notices (births, weddings, deaths, job offers). The journal also had a literary section and a section for reviews of books received by the college.

The journal sold advertising space for Huesca’s medical practitioners and pharmaceutical and orthopaedic products. Its only iconographic content consisted of just three photographs: Mr. Molanés, Provincial Health Inspector; Mr. Fernández Carril, in a feature after his election as Civil Governor of Huesca; and Mr. Antonio Sánchez García del Real, president of the National Federation of Colleges of Practicantes. Finally, there were also published, together with the newspaper, the collectable booklets *Legislación y Organización Sanitaria. Servicios Auxiliares Médicos*.

## Recurring topics covered over the years

### Relationship between *practicantes* and nurses

When nursing studies were officially created in Spain in 1915, *practicantes* were quick to voice their opposition. They felt that the new training would enable nurses to be attributed the same competences as them, endangering their professional future. The relationship between the two professions was therefore troubled from the outset by this accusation that nurses were trespassing on the professional territory of *practicantes*. In addition, nursing training took less time and was less expensive than the training required for *practicantes*. To qualify, nurses had to pass a theoretical and practical examination judged by a board of examiners and obtain a certificate issued by the dean of the Faculty of Medicine upon payment of 13 pesetas (Spain, 21 May 1915, 13 Sep. 1931). In contrast, *practicantes* were required to complete and pass the first three years of the baccalaureate, followed by two years of university studies (Spain, 5 Jan. 1935), at a total outlay of 323 pesetas (Pérez-Estalayo, 1915).

Some historians attribute this turbulent relationship to the ideological context of gender inequality at the time (Arroyo-Rodríguez et al., 2011; Calvo-Calvo, 2014). According to the prevailing discourse in Spanish society, to talk about *practicantes* and nurses was to talk about two different things. One book used for the teaching of these auxiliary medical professions reads as follows:

There is an enormous difference between a *practicante* and a nurse, and this must be emphasized so that the former can assert his rights.

The *practicante* is an expert, with a professional title, more limited in scope, but as respectable as any other official career, who carries out the prescriptions of the doctor ‘in accordance with science.’

The nurse is any person who does what the doctor orders, ‘in accordance with practice’ (García-Sierra, 1923, p.3-4; emphasis added).^2^

In other words, men were synonymous with power and culture; women, with submission and nature (Arroyo-Rodríguez et al., 2011). In contrast, in Anglo-Saxon countries, nursing was a profession that admitted both sexes; however, it had marked connotations determined by gender relations and the ideological designation of nursing as a woman’s job. Being associated with nursing would compromise a man’s prestige and social status within the patriarchal culture (Evans, 2004).

In 1904, legislation was passed that authorized women to train in and practise the profession of *practicante* (Spain, 12 Aug. 1904), even though no evidence has been found of any explicit prohibition of female *practicantes* prior to this date (Blázquez-Ornat et al., 2012). In fact, in the academic year 1900-1901, there were eight female students enrolled on the training course for *practicantes* (Capel-Martínez, 1982). Curiously, a woman did not need her husband’s permission to pursue such studies, but she did if she wished to pursue midwifery studies.

Twenty years after the official regulation of the nursing degree in Spain, the situation was still tense. Issue number 1 of *El Practicante Oscense* reports that on June 5, 1935, the *practicantes* sent a petition to the director-general of Health calling for him to establish which functions were their exclusive domain and which ones nurses could perform, without impinging on those of the *practicantes*’ work (Acta…, 1935). When a resolution to this impasse was not forthcoming, the *practicantes* took their claim to the Ministry of Public Instruction (Acta…, 1936). In the middle of the civil war, nurses’ functions were limited. *El Practicante Oscense* reported the Ministry of the Interior’s orders, issued on May 24, 1938, that performing the activities of a *practicante* was prohibited to all those who were not qualified as such (Ramiro el Lego, 1938; Spain, 10 June 1938).

### Payment of salaries and arrears

It was thanks to the journal *El Practicante Oscense* that we learned that one of the issues that most concerned the *practicantes* at the time was the receipt of their salaries. This subject was one to which a considerable number of pages was devoted.

To give some background, the Law of Sanitary Coordination, of July 11, 1934, was the main contribution by the Republic to Spanish sanitary legislation. This law sought to improve the organization and efficiency of health and charitable health services entrusted to the deputations and municipalities through provincial commonwealths of municipalities: administrative bodies under the Ministry of Labor, Health, and Welfare. Health care regulation was the public function of municipalities, provinces, and states in collaboration, under the technical and administrative direction of the state. Each province’s health workers – their general physicians, obstetricians, pharmacists, *practicantes*, midwives etc. – would nominate one person, or more, to receive the total amount allocated to their profession in the municipal budgets, creating a general payroll, which they would sign when they received the wages. The president of the Provincial Board of Full Physicians (Junta Provincial de Médicos Titulares) and the presidents of the official colleges of the other professions would, at the request of the president of the commonwealth of municipalities, summon the interested parties and submit the minutes appointing these representatives (Spain, 15 July 1934). *Practicantes* and midwives were incorporated into the republican health care model by means of a decree, passed on June 14, 1935, which established the Practicantes of Public Domiciliary Assistance Corp and Municipal Midwives Corp (Cuerpo de Practicantes de Asistencia Pública Domiciliaria y Cuerpo de Matronas Titulares Municipales). Their respective regulations clearly identified their functions (Spain, 19 June 1935). Previously, it had been the Corp of Full Physicians (Cuerpo de Médicos Titulares), that is, the physicians permanently in charge of the medical and surgical care of poor families in the municipalities, renamed Corp of Public Domiciliary Care Physicians (Cuerpo de Médicos de Asistencia Pública Domiciliaria), who suffered a similar problem with respect to the payment of their salaries.

The aim of the Law of Sanitary Coordination was to ensure that public health workers employed by municipalities received their full wages on time. This was because some of these professionals were owed several months’ wages. However, six months after the law came into force, only 10% of the municipalities had paid their respective public servants in full – a statistic that was denounced in the January 1936 issue of *El Practicante Oscense* (Naya-Casademont, 1936). According to another article published in the same issue, the municipalities were so fiercely opposed to this law that they even considered organizing a union of municipalities to get it repealed (Broto, 1936). To address this problem, and on the initiative of the Official College of Practicantes of Almería, the Consultative Board of Colleges of Practicantes convened in Madrid in December 1935 and subsequently submitted a bill that would have municipal health workers paid directly by the state (Federación…, 1936).

### Tools for socialization

From the outset, the assembly movement served as one of the pillars of the activities undertaken by the colleges of *practicantes*. In the interest of the *practicantes* from Aragón, *El Practicante Oscense*, in June 1936, called for an assembly of rural Aragonese *practicantes* to be held (Piquer-Lafuente, 1936; Piquer-Lafuente, Naya-Casademont, 1936). This would have been the second assembly of its kind, the first one having taken place in Zaragoza in May 1933, when 16 resolutions were passed, including the constitution of a regional federation of *practicantes* colleges (La Asamblea Regional…, 1933). However, plans for the second assembly were cut short by the outbreak of war.

### The professional association of practicantes in Huesca during the civil war

The Spanish Civil War broke out on July 18, 1936, dividing the country into two geographical zones: the National Zone, in the hands of the insurgent faction, and the Republican Zone, which remained loyal to the Republic. The colleges of *practicantes* were not untouched by this development, suffering different fates according to the zone in which they were situated. In the provinces loyal to the Republic, the trade unions took control of their respective colleges, which were soon dissolved. In the provinces that had rebelled, the colleges remained in place and endeavoured to re-establish national unity (Expósito-González, 2023).

The region of Aragón was a clear example of this disparate fate, because while the Nationalists controlled the three provincial capitals, the Republicans established control over the rural areas. The Official College of Practicantes of Huesca, whose membership was predominantly rural, was surprised by this situation, as very few of its members remained in the National Zone. After almost two years of siege by the Republican troops in their attempt to regain control, Huesca was “liberated”^3^ by the Nationalists on March 24, 1938. *El Practicante Oscense*, which resumed publication in July 1938, two years into the war, headed its first editorial with the cry of “Up Spain!” Its contents and tone reflected the recent historical period: “Now that life in this Capital has been normalized, since its heroic siege, and the province has been liberated by our Glorious and Invincible Army, *El Practicante Oscense* has reappeared with the enthusiasm of always” (Naya-Casademont, 1938b, p.1).

Even when Huesca was on the front line of the war, it stayed in contact with colleges from the rearguard. Once Franco’s government was installed in Burgos, the college called a meeting of college presidents to form the Provisional National Board of Colleges of Practicantes of Spain, which was attended by Pascual Naya as the representative of Huesca. There were some points of contention between the newly created board and the college of Huesca. One dispute that was reported in *El Practicante Oscense* was over the failure of the Huesca college to pay the fees it owed to the national organization, as had been determined in its previous meeting (Disciplina…, 1938).

As the war progressed and more Spanish towns were liberated, the victory of the Nationalists became an open secret, and the principles of the future state were designed in accordance with the theses of National Syndicalism. For the class of *practicantes*, this heralded a new orientation: a Health Union. In an extraordinary general meeting, the *practicantes* of Huesca voted en bloc to join the Technical-Sanitary section of FET de las JONS (Acta…, 1938). In a climate of political purges, the governing board of the Huesca college was put in charge of processing the files that would determine which *practicantes* would lose their jobs (Naya-Casademont, 1938a).

## Final considerations

*El Practicante Oscense*, the journal published under the auspices of the Official College of Practicantes of Huesca and its province, was created a few months before the tragic outbreak of civil war in Spain, a period of great social and professional unrest for *practicantes*. Although modest, the journal was typical of the kinds of publication created by and for professional groups at the time. Its purpose was to defend the interests of the professional class, especially those who worked in rural areas and were faced with serious problems, such as the employment of unqualified people to work as *practicantes* or the non-payment of wages. In addition, the journal helped forge the *practicante* identity by rejecting any link to the domestic care associated with nurses.

Despite the adverse labour situation, the *practicantes* from Huesca encouraged their Aragonese colleagues to hold a regional assembly to unite and face their problems together, which would give them a greater chance of success.

The preservation of this journal in its entirety allowed us to answer certain questions about the history of the organization and functioning of the colleges of *practicantes* during the Spanish Civil War. *El Practicante Oscense* suspended its publication at the beginning of the war. When it resumed publication, after the rebel troops took control of the province, the governing board of the college to which it was affiliated realigned itself with the Nationalist faction. From that moment on, it collaborated with Franco’s authorities in the political purges of *practicantes*.

The main limitations of this study are the scant information provided in each issue due to their limited size and the fact that the journal was provincial in scope. Future research could scrutinize the impacts of this and other journals of the same kind on the professional practices of the intended readership.

## Data Availability

Not deposited in a data repository.

## NOTES

1 We were unable to determine whether this issue was published or whether there was a typographical error in the numbering that was carried over to all the subsequent issues of the journal.

2 This and other citations of texts in Spanish were translated by the authors.

3 A term used to refer to the incorporation of territory by the rebel faction.
